# Spatial Inequalities and Influencing Factors of Self-Rated Health and Perceived Environmental Hazards in a Metropolis: A Case Study of Zhengzhou City, China

**DOI:** 10.3390/ijerph19127551

**Published:** 2022-06-20

**Authors:** Hongbo Zhao, Li Yue, Zeting Jia, Lingling Su

**Affiliations:** Key Research Institute of Yellow River Civilization and Sustainable Development & Collaborative Innovation, Center on Yellow River Civilization Jointly Built by Henan Province and Ministry of Education, Henan University, Kaifeng 475001, China; 10340024@vip.henu.edu.cn (H.Z.); jiazeting@henu.edu.cn (Z.J.); 10340058@henu.edu.cn (L.S.)

**Keywords:** spatial inequality, self-rated health, perceived environmental hazard, geographical context, Zhengzhou City

## Abstract

Research on environmental pollution and public health has aroused increasing concern from international scholars; particularly, environmental hazards are among the important issues in China, focusing public attention on significant health risks. However, there are few studies concentrated on how perceived environmental hazards are characterized by spatial variation and on the impact of these risks on residents’ health. Based on a large-scale survey of Zhengzhou City in 2020, we investigated how the self-rated health of residents and the environmental hazards perceived by them were spatially inequal at a fine (subdistrict) scale in Zhengzhou City, China, and examined the relationship among self-rated health, environmental hazards, and geographical context. The Getis–Ord Gi* method was applied to explore the spatially dependent contextual (neighborhood) effect on environmental health inequality, and the ordered multivariate logistic regression method was used to examine the correlative factors with environmental hazards, geographical context, and health inequality. The results reveal that self-rated health and environmental hazards were disproportionately distributed across the whole city and that these distributions showed certain spatial cluster characteristics. The hot spot clusters of self-rated health had favorable environmental quality where the hot spot clusters of environmental hazards were located and vice versa. In addition, health inequality was evident and was related to gender, income level, educational attainment, and housing area of residents, and the inequalities of environmental hazards existed with respect to income and housing area. Meanwhile, environmental risk inequalities associated with the social vulnerability of residents (the poor and those with low educational attainment) were obvious, with those residents experiencing a disproportionately high exposure to environmental hazards and reporting bad health conditions. The role of the geographical context (subdistrict location feature) also helps to explain the spatial distribution of health and environmental inequalities. Residents with better exposure to green coverage generally reported higher levels of self-rated health condition. In addition, the geographical location of the subdistrict also had a significant impact on the difference in residents’ self-rated health status. The purpose of this study is to provide reference for policy makers to optimize the spatial pattern of urban public services and improve public health and environmental quality at a fine scale.

## 1. Introduction

During the last few decades, China has undergone fast industrialization and urbanization [1,2], and the urbanization rate of China’s permanent population exceeded 60% in 2020 (http://www.stats.gov.cn/, accessed on 26 May 2022). Continued development has caused a series of environmental issues and has seriously threatened public health [3,4]. The annual disease burden caused by the environment and its related factors in China is 21%, which is 8% higher than that in the United States [5]. Although some pollution control measures have been taken in recent years, air pollution and other environmental pollutants are still the main environmental health problems in China [6,7]. Additionally, rapid urbanization and extensive economic growth have also generated a series of other environmental hazards that cause grave risks to residents’ health, including water pollution, soil heavy metals, industrial solid waste, etc. [8,9,10].

For a long time, environmental hazards and human health issues have attracted attention around the world [11,12,13,14]. These related research studies can be divided into two major parts: Firstly, the spatial patterns of environmental pollution and health indicators (such as various disease morbidity, mortality, etc.) have been studied at macro regional scales [15,16,17]. These studies concentrate on the dose–response relationship between different environmental factors and human health and provide the basis for formulating environmental hygiene standards and preventive measures at the macro level [18,19]. Secondly, other studies have paid attention to the socially disadvantaged groups (children, the poor, the aged) who undertake unequal costs with respect to the harmful environmental impacts, such as industrial waste, exhaust emissions, and air pollution [20,21,22]. These research studies generally extend their analyses to gain insight into environmental justice and health inequality. For example, through the empirical analysis of the dynamic changes in air quality in Britain from 2001 to 2011, Mitchell et al. [23] found that the improvement of air quality was the most obvious in affluent areas, while the decline in air quality in deprived areas was evident; this situation further aggravated the environmental injustice and health inequality in Britain. In general, these previous studies indicate that the adverse effects of environmental hazards are characterized by an unbalanced distribution at the social, economic, and demographic levels, and disadvantaged groups (poor people, the elderly, migrants) bear more health risks and harm caused by environmental hazards. However, the relationship between environmental hazards and health inequality has rarely been noticed in developing countries [24]. Especially in China, the impacts of environmental hazards on resident health inequality have largely been neglected at the intracity scale [25]. In order to develop more just environment planning measures and health policies, more research needs to investigate the complex relationship between health inequality and environmental hazards at the micro scale in different social and spatial contexts [26].

In recent years, the impact of public exposure to environmental pollution on health has been a subject of concern and has urged to use resident survey data drawn from small samples or multicity times-series observations [27,28,29]. For example, Lu et al. [30] found that ambient air pollution (PM_2.5_, PM_10_, and SO_2_) and water pollution had significant deleterious effects on physical health and mortality. Other studies have also investigated the spatial patterns of environmental hazards and have revealed that the detrimental effects of environmental pollution are unevenly distributed across socioeconomic strata [28,31]. Furthermore, Holdaway also argued that environment-related health risks should be analyzed in China by using geographical methods to understand which population groups are more vulnerable [32].

In order to further measure the relationship between environmental hazards and health inequality, some scholars have divided environmental hazards into *subjective* or *objective*, where subjective refers to residents’ perception of environment hazards, while *objective* refers to the occurrence of exposure to environmental pollution [33,34]. Although residential proximity to environmental hazards is widely used in environmental health research [35], objective analyses of environmental hazards are not possible because of fine-scale data being constrained in China. In addition, studies have shown that subjective measures can accurately reflect the long-term status of public exposure to environmental hazards, which has been considered as important as objective assessment in reflecting public health conditions [28,34,36]. Since individual subjective data may more truly reflect residents’ environmental hazard perceptions and health conditions, they should be used to better renovate and improve the urban environment based on people’s needs. We utilized the subjective evaluation method to explore the relationships between health conditions and environmental hazards in Zhengzhou City. The exploration of the relationships between environment hazards and health is informative to develop Chinese environmental governance and public health policies. Meanwhile, understanding the spatial patterns of environmental hazards is very important for comprehending health inequality in the city and helping to formulate relevant environmental control and public health policies for specific places in China. 

To address the existing research gap, this study focused on an intracity case to investigate the spatial patterns of environmental hazards and residents’ health at a fine scale, based on a large-scale survey of Zhengzhou City in 2020. In this research study, we explored the relationships between environmental hazards perceived by residents and their self-rated health. In addition, the geographical context was analyzed to better understand how various geographical attributes can impact environmental health inequality. Methodologically, a cold–hot spot analysis was applied to examine the spatially contextual (neighborhood) effect on environmental health inequality by using the Getis–Ord Gi* model. In addition, we investigated the subjective measurement of the four main perceived environment hazards, including air pollution, noise pollution, water pollution, and landfill pollution. Considering the structural characteristics of the data, the relationships between perceived environmental hazards and self-rated health were examined using the ordered multivariate logistic regression model. The results of this study reveal the relationships between environmental hazards and health inequality at a fine scale in a Chinese megacity, and the importance of the geographical context on environmental-health risks is here explored in the hope of better informing the formulation of public health policies.

## 2. Materials and Methods

### 2.1. Study Area

**Figure 1:**
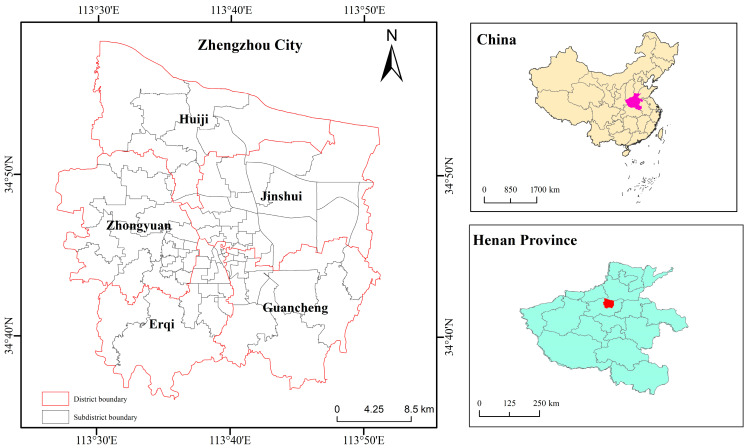
The location of Zhengzhou City.

Zhengzhou City is the capital of Henan Province, and it is an important central city and transportation hub in central China (Figure 1) located in the middle and lower reaches of Yellow River (112°42′ E–114°14′ E, 34°16′ N–34°58′ N). According to the Zhengzhou Statistical Information Net (http://tjj.zhengzhou.gov.cn, accessed on 26 May 2022), at the end of 2020, the area under the city’s administration was 7567 km^2^, and its built-up area was 709.69 km^2^. The total population of Zhengzhou City was 12.6 million. Similar to many other megacities in China, Zhengzhou City has experienced rapid urbanization and population growth in the past several decades. From 1978 to 2020, the urbanization rate of Zhengzhou City increased from 32.4% to 78.4%, and the population density increased from 588 people per km^2^ to 1694 people per km^2^ (http://tjj.zhengzhou.gov.cn/ndsj/3134558.jhtml, accessed on 26 May 2022). Such dramatic urbanization process inevitably resulted in the deterioration of the urban environment and produced a series of pollution problems. These environmental hazards have an adverse effect on residents’ health conditions.

### 2.2. Data Sources

Our analysis draws on a large-scale residential perception and health survey of Zhengzhou City in 2020. The purpose of the survey was to evaluate resident’s health conditions and perception of their surrounding environment. Only residents who had lived in Zhengzhou City for more than one year and had reached the age of 18 could meet the requirements of this survey. The combination strategy of quota sampling and stratified random sampling was adopted, and eight Districts of Zhengzhou City (Jinshui, Zhongyuan, Erqi, Guancheng, Jingkai, Huiji, Gaoxin, and Zhengdong New District) were selected as our survey fields. In total, 4075 questionnaires were returned, and about 3842 were valid. Further details of the survey are provided in Table 1.

According to the questionnaire, this study extracted three aspects of variables to explore the impact on residents’ health. The first category included a range of individual sociodemographic characteristics, mainly including gender, age, monthly income, education, marital status, residence status, housing type, etc. These variables are thought to be related to health inequality. Furthermore, four perceived environmental hazards were selected to examine the impact on residents’ health conditions, including air pollution, noise pollution, water pollution, and landfills. Finally, a series of geographic context variables, including walking distance to the nearest hospital, green coverage, urban waterlogging, and the location of each subdistrict, were analyzed. A comprehensive consideration of these variables can contribute to a better understanding of the relationships between perceived environmental hazards and the self-rated health of residents.

### 2.3. Methods

**Figure 2:**
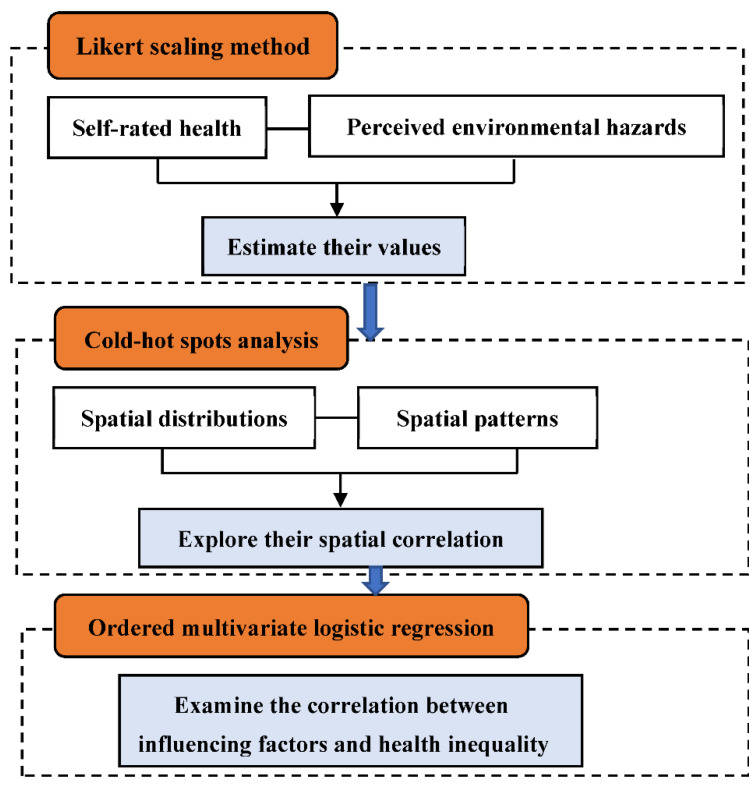
The method framework in this study.

In this study, the method framework combining Likert scaling, cold–hot spots, and ordered multivariate logistic regression model is presented in Figure 2.

#### 2.3.1. Likert Scaling

The Likert scaling method usually adopts the form of a 5-point scale, ranging from 1 (very good) to 5 (very bad) [37]. It reflects the responders’ comprehensive attitude towards the survey topics. In this study, the self-rated health of residents based on a Likert scale was used to reflect the overall health condition. They were asked the question as follows: How would you assess your overall health condition in the last half year? The respondents used a 5-point Likert scale to select their health status, ranging from 1 (very good) to 5 (very bad). Due to the objective data being unavailable at a fine (subdistrict) scale in Zhengzhou City, four kinds of perceived environmental hazards (air pollution, water pollution, noise pollution, and landfills) were evaluated by 5-point Likert scales ranging from 1 (very high) to 5 (very low); the answers assessed the exposure to the four types of environmental hazards in their neighborhood. Other variables in our analysis, including walking distance to the nearest hospital, green coverage, and urban waterlogging, were also evaluated using the Likert scale to assess the responders’ evaluation of their neighborhood.

#### 2.3.2. Cold–Hot Spot Analysis

In this study, in order to explore the spatially dependent contextual (neighborhood) effect on health inequality, the Getis–Ord Gi* method was used to measure the cold–hot spots of environmental hazards and health status. A cold–hot spot analysis can identify the location where the high values or low values of self-rated health and environmental hazards are clustered in space, indicating that they are affected by some spatial factors and have spatial correlation. The hot spots mean that self-rated health or environmental hazards themselves should have high values and that their surroundings are high values, that is, the area is a gathering area of high values. On the contrary, the cold spots indicate that not only their own values are very low, but their adjacent values are also all low values, that is, the area is a gathering area of low values. ArcGIS 10.2.2 software (Environmental Systems Research Institute, Inc., RedLands, CA, USA) was employed to divide the perceived environmental hazards and self-rated health into four levels: cold spot zones, sub-cold spot zones, sub-hot spot zones, and hot spot zones. The Getis–Ord Gi* method is expressed by the following equation [38]:

(1) $$G_{i}^{*}(d)=\frac{\sum_{j=1}^{n}W_{ij}(d)X_{j}}{\sum_{j=1}^{n}X_{j}}$$

where *X_j_* denote the value of variate *j*, and *W_ij_*(*d*) denotes the spatial weight matrix between *i* and *j*. The standardized Getis–Ord model is evaluated by the significance of the Getis–Ord statistic [39]. The standardized version of the Getis statistic is:

(2) $$Z(G_{i}^{*})=\frac{G_{i}^{*}-E(G_{i}^{*})}{\sqrt{Var(G_{i}^{*})}}$$

where *E*(G_i_*) is the expected value of G_i_* and *Var* (G_i_*) is the variance value of G_i_*. If *Z*(G_i_*) > 0, this shows that the spatial clustering of values belongs to hot spot zones. If *Z*(G_i_*) < 0, this shows that the spatial clustering of values belongs to cold spot zones.

#### 2.3.3. Ordered Multivariate Logistic Regression Model

Ordered multivariate logistic regression is one of the common statistical methods used to examine the influence of complex factors in social science analysis [40]. In this study, because the variables had the characteristics of ordered multivariate, this model was applied to examine the correlation of the above-mentioned factors with environmental hazards and health inequality. *X* = (*x*_1_, *x*_2_, …, *x_m_*) denotes the independent variable vector, and *Y* denotes the multivariate ordered dependent variable. $\pi_{j}=P(Y=j|X),j=1,2,\ldots ,n$, $\sum_{j=1}^{n}\pi_{j}=1$, where $P_{j}$ denotes the estimated value of $\pi_{j}$. The model is expressed by the following equation [41]:

(3) $$p_{1}=\exp\left[a_{1}+\sum_{i=1}^{m}b_{i}x_{i}\right]/\left[1+\exp[a_{1}+\sum_{i=1}^{m}b_{i}x_{i}]\right]$$

(4) $$P_{j}=\frac{\exp\left[a_{j}+\sum_{i=1}^{m}b_{i}x_{i}\right]}{\left[1+\exp[a_{j}+\sum_{i=1}^{m}b_{i}x_{i}]\right]}-\frac{\exp\left[a_{j-1}+\sum_{i=1}^{m}b_{i}x_{i}\right]}{\left[1+\exp[a_{j-1}+\sum_{i=1}^{m}b_{i}x_{i}]\right]};j=2,3,\ldots ,k-1$$

(5) $$P_{k}=1-\exp\left[a_{k-1}+\sum_{i=1}^{m}b_{i}x_{i}\right]/\left[1+\exp[a_{k-1}+\sum_{i=1}^{m}b_{i}x_{i}]\right]$$

where *x_i_* denotes the explanatory variable; *a_i_* denotes the estimate of the model intercept; *b_i_* denotes the estimated value of the regression coefficient; *m* denotes the number of variables; and *k* denotes the level number, ranging from 1 to 5, and $P_{k}$ denotes the explained variable.

## 3. Results

### 3.1. The Assessment of Self-Rated Health and Environmental Hazards

**Figure 3:**
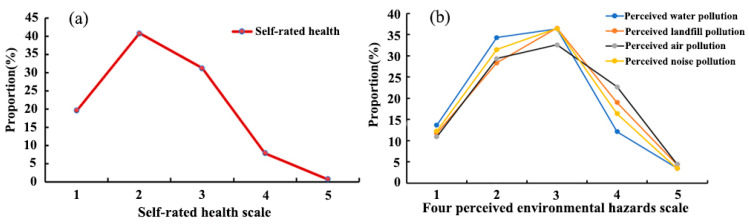
Proportion (%) of residents in self-rated health (**a**) and four perceived environmental hazard types (**b**).

**Figure 4:**
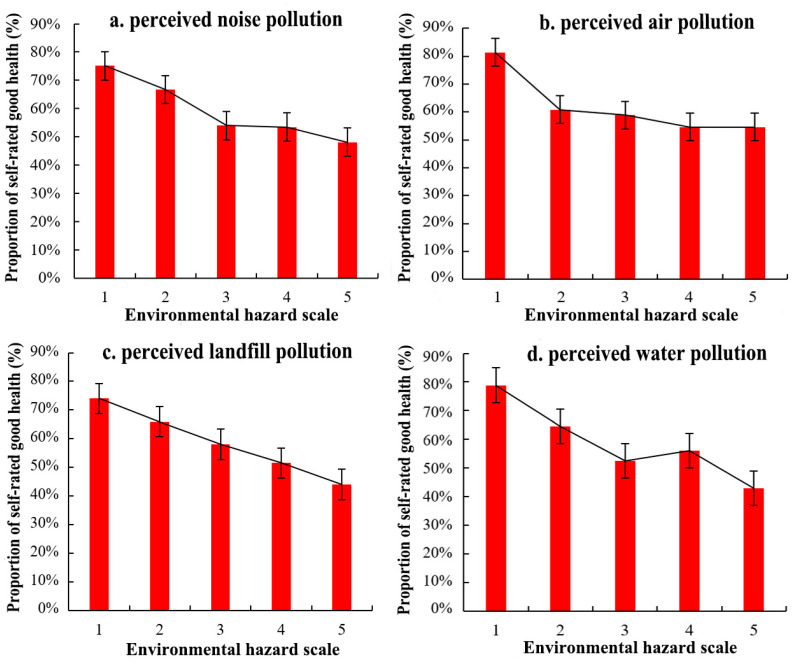
Proportion of residents reporting self-rated good health by four perceived environmental hazard types ((**a**) noise pollution, (**b**) air pollution, (**c**) landfill pollution, (**d**) water pollution).

Figure 3a shows the proportion of respondents with respect to self-rated health and to perceived exposure to four environmental hazards (Figure 3b). After calculation, the mean self-rated-health value of residents was 2.29. In general, most residents assessed their health status as good, with 19.55% and 40.76% of residents reporting their health status as very good or good, respectively, while 7.86% and 0.65% residents evaluated their health condition as bad or very bad, respectively. Additionally, there was an obvious variation between the proportion of the perceived exposure to four environmental hazards. In total, 15.64% and 19.83% of residents reported their perceived water pollution and noise pollution as poor, while there were 23.37% and 27.15% of residents reporting bad perceived air pollution and landfill pollution, respectively. It indicates that many residents considered that air pollution and landfill pollution were more serious than noise and water pollution in Zhengzhou City. The proportion of residents reporting good health conditions at different grades of exposure to four types of environmental hazards is shown in Figure 4. In general, the probability of assessing a good health status gradually decreased with the increase in residents’ perceived severity of exposure to environmental hazards in the study area. This characteristic is relatively obvious with perceived landfill pollution.

### 3.2. Spatial Distribution Characteristics of Self-Rated Health and Environmental Hazards

**Figure 5:**
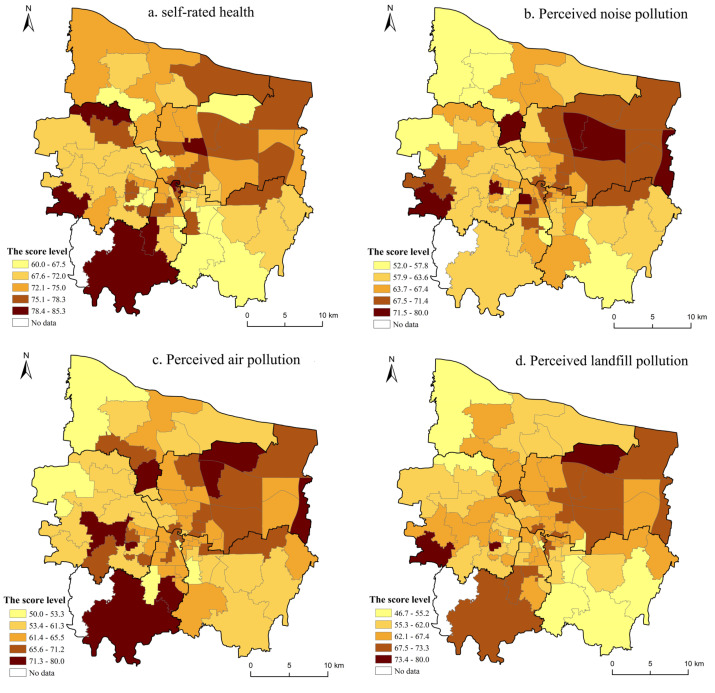

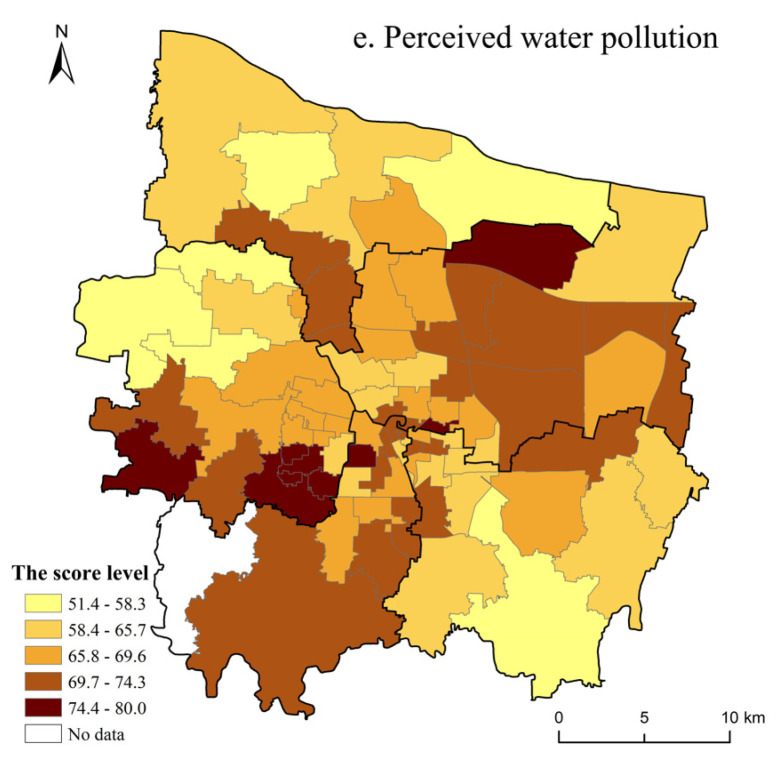
Spatial pattern of self-rated health (**a**) and four perceived environmental hazards (**b**–**e**) at the subdistrict (jiedao) level of Zhengzhou City.

A spatial analysis was subsequently conducted using ArcGIS10.2.2 software (Environmental Systems Research Institute, Inc., RedLands, CA, USA) to demonstrate the spatial pattern characteristics of self-rated health and perceived exposure to environmental hazards at the subdistrict (jiedao) scale in Zhengzhou City (Figure 5). In order to facilitate the spatial analysis, we converted the 5-point scale to a 0–100 scale for practical purposes. The higher the score of self-rated health was, the better the health status in the subdistrict, while the lower the score was, the worse the health status in the subdistricts. When the scores of each category of perceived environmental hazards were higher, it indicated lower pollution in the subdistrict, while when the score was lower, it indicated more serious pollution.

Overall, self-rated health and four perceived environmental hazards showed significant spatial inequality features. Figure 5a indicates the spatial distributions of the self-rated health scores in each subdistrict in Zhengzhou City and demonstrates the clustering of the subdistricts with a lower proportion of good health in the west and south of the city, while a higher proportion of good-health clusters is evident in the eastern and northern areas. The reasons for the differences are urban expansion and industrial development, which have led to a worsening of the living conditions in the southern area. The Moran’s I (spatial autocorrelation) value of the self-rated health status was 0.130 (*p*-value < 0.05), suggesting the spatial dependence of self-rated health among subdistricts. Figure 5b illustrates the spatial distributions of perceived noise pollution in each subdistrict in Zhengzhou City and indicates the clustering of subdistricts with serious noise pollution in the southern and northwestern areas. This can be explained by the reason that noise generated by urban construction and enterprise production affects the lives of residents. The Moran’s I (spatial autocorrelation) statistic of the perceived noise pollution was about 0.201 (*p*-value < 0.05), suggesting spatial dependence among subdistricts. Figure 5c shows the spatial characteristics of perceived air pollution in each subdistrict of Zhengzhou City and manifests the clustering of subdistricts with high score levels of perceived air pollution in the southwest and northeast of the city. The Moran’s I (spatial autocorrelation) statistic of the perceived traffic-related air pollution was about 0.065 (*p*-value = 0.123), indicating that there was no spatial dependence among subdistricts. Figure 5d reveals the spatial distribution characteristics of perceived landfill pollution in each subdistrict of Zhengzhou City and indicates the clustering of subdistricts with serious landfill pollution in the surrounding areas near the central city, southern areas, and northwestern area. The Moran’s I (spatial autocorrelation) statistic of the perceived landfill pollution was about 0.203 (*p*-value < 0.05), indicating spatial dependence among subdistricts. Figure 5e displays the spatial patterns of perceived water pollution in the subdistricts of Zhengzhou City and indicates the clustering of subdistricts with serious water pollution in the southeastern and northern areas. This is because industrial production emits large amounts of industrial wastewater and seriously affects water quality in dwelling zones. The Moran’s I (spatial autocorrelation) statistic of the perceived water pollution was about 0.206 (*p*-value < 0.05), indicating spatial dependence among subdistricts.

### 3.3. Cold–Hot Spot Analysis

**Figure 6:**
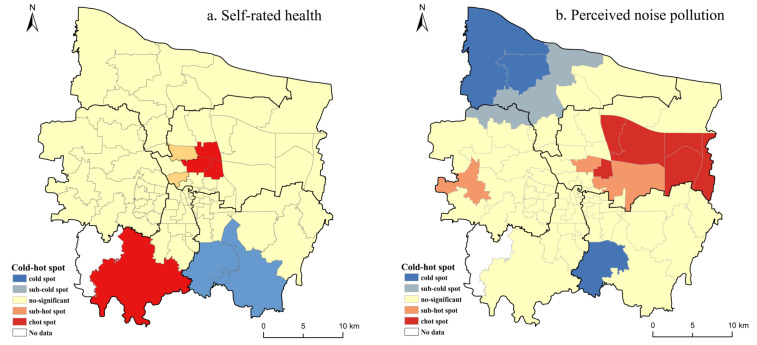

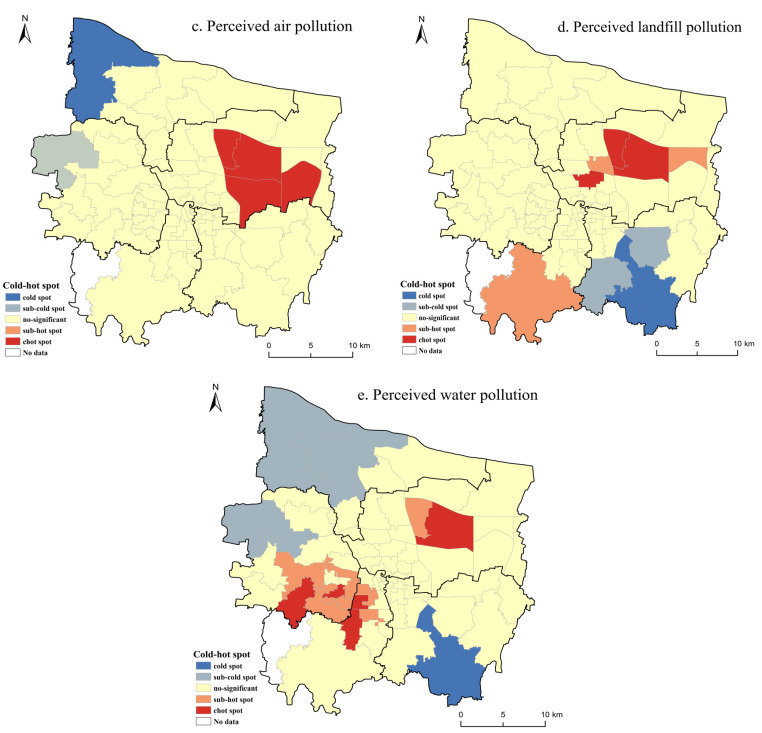
The cold–hot spot patterns of self-rated health (**a**) and four perceived environmental hazards (**b**–**e**) at the subdistrict (jiedao) level of Zhengzhou City.

The spatial cold–hot spot patterns of self-rated health and perceived exposure to four environmental hazards in the subdistricts of Zhengzhou City are shown in Figure 6. Large clusters of self-rated-health hot spots appeared in the northern and southwestern parts of the city center, and only a few small clusters of cold spots appeared in the southeastern part of the city (Figure 6a). We observed that the cold spots of four perceived environmental hazards were prominently distributed in the urban fringe, mostly in the northwestern and southern parts of the city (Figure 6b–e). These finds can be interpreted with the knowledge that these areas have relatively more industrial parks and worse environmental quality. Nevertheless, a hot spot cluster could be found in the northeastern and western areas of Zhengzhou City. These areas are mainly distributed with green park space, and the environmental quality is relatively good. Overall, the hot spot clusters of self-rated health across the entire city showed favorable environmental quality where hot spot clusters of environmental hazards were located and vice versa. This indicates that there was a certain correlation between the health status of residents and the surrounding environment.

## 4. Factors Influencing Health Inequality

### 4.1. Self-Rated Health and Sociodemographic Characteristics

The estimation results from the ordered multivariate logistic regression model indicate that some of the sociodemographic variables were obviously correlated with self-rated health in Zhengzhou City (Table 2). As model 1 in Table 2 shows, there was a significantly negative relationship between the female group and their self-rated health, which indicates that women thought their own health status was better than men’s health status. However, there were no significant correlations between residents’ age and self-rated health in the study area. Regarding education level, there was a significant correlation between education level and self-rated health status, which shows that people with an advanced degree reported better health status than people with low-level education. Compared with married residents, residents with other marital statuses (divorced or widowed) had worse health conditions, while unmarried residents had a better self-rated health level. A significantly positive effect on health was found for the income level, which indicates that high-income people tended to have a good health status. This result is consistent with existing studies that proved a significant effect of household income level on self-rated health in China [27]. We also found that the residence status and housing type factors were not significantly associated with self-rated health. In terms of the housing areas, we found that residents living in a small house were significantly more associated with a poor health status than their counterparts. In contrast, people living in a big house significantly tended to report a good self-rated health status.

### 4.2. Self-Rated Health and Perceived Environmental Hazards

To estimate the relationship between the health status and environmental hazards, we included four environmental hazard variables in our regression. As model 2 in Table 2 shows, all the four perceived environmental hazards were revealed to be markedly related to self-rated health conditions, with those who perceived lower exposure to environmental hazards (noise pollution, air pollution, landfill pollution, and water pollution) being more likely to suggest good health conditions. For example, compared with the very low level of perceived noise pollution, residents who reported a higher level of noise pollution commonly reflected worse self-rated health status. Among these four environmental hazards, the coefficients of water pollution and noise pollution were relatively larger, indicating that these two factors had greater impacts on resident’s self-rated health status. It is noteworthy that the estimates of resident’s sociodemographics were still significantly correlated with self-rated health, including gender, marital status, housing area, and other factors. These findings demonstrate that neighborhoods with lower exposure to environmental hazards can improve residents’ health status and alleviate the health inequality of social and economic characteristics of different groups [42,43].

### 4.3. Self-Rated Health and Geographical Contextual Effect

Regarding locational variables, the walking distance to the nearest hospital was significantly associated with the self-rated health status, which suggests that people residing in neighborhoods close to hospitals had statistically higher odds of reflecting good self-rated health status. Especially, people living in neighborhoods more than 3 km away from hospitals had a more significant relationship with self-rated health. This result indicates that living near hospitals might demonstrate the convenience of medical treatment in case of illness, which could provide residents with psychological comfort and enhance the probability of good self-rated health conditions. In addition, green coverage was also found to be significantly related to residents’ health status. Compared with a good level of green coverage, people who perceived a bad level of green coverage reported poorer self-rated health conditions. This indicates that increasing green coverage can effectively improve the health level of residents [44,45]. In this study, we also considered the impact of urban waterlogging on residents’ health. The findings show that when urban waterlogging was worse, residents reported poorer self-rated health.

The impact of the geographical location on self-rated health was quantified by adding the subdistrict locational variable to the regression model (Table 2). Because there were 81 subdistricts in this study, considering the limited space, we cannot list them one by one. As model 3 in Table 2 shows, the subdistrict locational variable was found to be significantly related to self-rated health, suggesting a geographical heterogeneity effect across the subdistrict scale. This indicates that the chance of self-rated good health would increase if individuals were moved from subdistricts with poor environmental quality to subdistricts with a good environment. Additionally, Figure 5 also confirms the geographical pattern whereby a relatively higher score of self-rated health in suburban areas coincided with a better level in terms of environmental hazards, whereas a lower score of self-rated health in southern and northwestern areas coincided with worse perceived environmental hazards in Zhengzhou City.

## 5. Discussion

### 5.1. Geographical Distributions of Self-Rated Health and Perceived Environmental Hazards

Based on the above quantitative analysis, the findings indicate that self-rated health and the four perceived environmental hazards were characterized by an unbalanced distribution at the subdistrict scale in Zhengzhou City. In general, people resident in subdistricts in the central and southern areas had lower levels of self-rated health, while there was a higher proportion of good-health clusters in the eastern and northern areas. Similar to the pattern of health inequality in Zhengzhou City, other studies also found a similar distribution of health inequality in Chinese cities, reporting a relatively low value of self-rated health in urban core areas [25]. Regarding the perceived environmental hazards, subdistricts with higher exposure to air pollution and noise pollution were revealed in the northwestern and southern areas of Zhengzhou City, probably because the traffic congestion in these areas is more serious than that in other areas. Subdistricts with more landfills and water pollution are mainly spread in the northern and southern marginal areas of Zhengzhou City, because most manufacturing industries are distributed in these districts. Our findings also indicate a clear relationship between self-rated health and perceived environmental hazards, that is, the clusters of good self-rated health across the entire city showed favorable environmental quality where high levels of environmental hazards were located, which is consistent with the findings in other studies [28,34].

Our study also investigated health inequality and environment hazards across the socioeconomic features of residents in Zhengzhou City. First, differences in health conditions were revealed with respect to gender and marital status: Females had a higher proportion of self-rated good health, while males had a significantly lower proportion of self-reported good health. This is because with the rapid economic development, women pay more and more attention to their own health status, while men bear greater responsibility and pressure, resulting in their poor perception of health status. Compared with married residents, the unmarried status of residents corresponded with better health conditions. Second, different income levels of residents also showed obvious variations. A lower income level of residents corresponded to poorer self-reported health, while people with higher income reported higher self-rated health, which is consistent with the research results found in other literatures reporting that the poor health status of low-income residents is associated with lifestyle, and living and working conditions [46]. Third, educational attainment also showed a noteworthy influence, whereby people with good educational background had higher odds of self-rating good health than those with primary education; this finding is consistent with previous studies [25]. In addition, people living in large-housing areas were significantly associated with a higher proportion of reporting good health, while people living in small-housing areas obviously had a lower proportion reporting good health. The reason can be explained by the fact that people living in large-housing areas have sufficient economic capacity to avoid high exposure to environmental hazards than those living in small-housing areas. Conversely, the poor live in small houses and do not have enough money to take full care of their health.

Concerning environmental hazards, which have a negative influence on residents’ health to some extent, we found that socially vulnerable groups reported higher exposure to environmental hazards (air, noise, water, landfills) than others. The perceived high exposure to four environmental hazards was biased toward low-income groups; the reason can be attributed to the living conditions of low-income groups, who generally dwell in lower-quality and less expensive housing near sources of environmental pollution. This result supports other existing findings showing that dwelling districts with lower socio-economic status (SES) generally have more adverse health effects caused by environmental hazards than affluent dwelling districts [43,47].

Our study investigated the geographical pattern of self-rated health and four perceived environmental hazards at finer scales to explore their cold–hot spots. Relatively high scores of self-rated health were located predominantly in the southwestern and northern parts of the city center, termed hot spots, and lower values areas appeared in the southeastern part of the city, termed cold spots. This study emphasizes the role of the surrounding environment, which influences the distribution of the health status of residents. We here establish the relationship between self-rated health and geographical context, with better exposure to green coverage resulting in higher levels of self-rated health conditions, while perceived worse urban waterlogging caused poorer self-rated health conditions. In addition, we also reveal that environmental inequality exists in Zhengzhou City, namely, social disadvantaged groups, including low-income people, experience disproportionately high environmental hazards.

### 5.2. Strengths and Limitations

In general, the lack of basic survey data leads to unclear information on the spatial pattern of resident’s health effects caused by environmental hazards at fine scales [25,48]. Our research study provides a research perspective to reveal the spatial distribution of self-rated health and its association with environmental hazards at the subdistrict scale in Zhengzhou City. Our study also develops a cold–hot spot model to analyze the spatial pattern characteristics of health inequality and environmental hazards. The spatial information of self-rated health and environmental hazards at fine scales provides a strong tool to identify “hot spots” of residents with a higher level of health status suffering from lower environmental quality. The results show that the spatial identification of cold–hot spots can recognize health inequality and optimize the resource allocation of public service facilities to reduce exposure to environmental risks. The geographical context effect on self-rated health is also revealed by using the ordered multivariate logistic regression model, and the importance of the geographical context in understanding health inequality and environmental hazards is proved in this study.

Our study is based on self-rated-health scores from individual-level data, and the perception factors of residents’ health should not be ignored in health promotion and environmental governance [49,50]. In fact, understanding residents’ perceptions of environmental risks helps to promote communication between the public and policy makers, to accurately identify health risks, and to further prioritize mitigation options [51]. Additionally, public participation is conducive to the improvement of China’s environmental protection regulations and reduction in environmental violations. Nevertheless, in our analysis, due to the lack of objectively measured data on residents’ health and environmental hazards, it was necessary to adopt a comprehensive method to verify the influencing factors of residents’ health. This indicates that collecting objective data on residents’ health and environmental hazards can better support public health promotion and environmental risk control in the future. Given the static nature of the subjectively evaluated data in this study, we may have overestimated the relationship between residents’ health and environmental hazards due to the lack of objectively measured data in this research study. So, our analysis is challenging, and it is necessary to use a combination of subjective and objective data on health and environmental hazards in future work.

### 5.3. Implications

In recent years, the Chinese government has gradually changed its economic development model and has taken strong measures to protect environmental quality, and remarkable results have been achieved in environmental governance [52]. Despite the encouraging improvements in the overall environment quality, environmental inequality still exists in cities. As per our analysis based on Zhengzhou City, socially vulnerable groups (the poor, low-educated people) experience a disproportionately high exposure to environmental pollution and worse health conditions, and these inequalities are contrary to the egalitarian principle of social development. Therefore, it is important to consider some environmental management measures to adequately protect the most disadvantaged groups. (1) Policy makers establish the integration of environmental protection, public health, and social justice into China’s governmental management system, and they fully understand the objectivity and seriousness of the existence of environmental and health inequalities in different social groups. (2) Urban planners should consider increasing green space and improving the health needs of residents in low-density green spaces. (3) Considering the importance of the layout of public facilities to residents’ health, it is necessary to improve the supply capacity and accessibility of urban public service facilities. (4) It is important to protect the public interests of urban socially vulnerable groups, i.e., by improving their living conditions and increasing the housing security of low-income residents. In summary, we hope these implications can provide a theoretical basis to support government managers in formulating more effective environmental protection and public health promotion policies.

## 6. Conclusions

Our research study used data from a large-scale questionnaire survey conducted in 2020 to develop a fine-scale analysis of self-rated health and environmental hazards in Zhengzhou City, which is a typical Chinese megacity with rapid urbanization. We also examine the role of socio-economic characteristics and geographical context in the relationships between health status and environmental hazards within urban areas. Our results demonstrate that self-rated health and perceived environmental hazards were unevenly distributed in the entire city and “cold-hotspots” of inequalities were spatially identified. Health inequality existed with respect to gender, income, educational level, and housing area. In addition, a clear finding is that the inequalities of environmental risks were obviously associated with the social vulnerability of residents (the poor and those with low-educational level), who experienced uneven exposure to four environmental hazards. We also indicate that residents’ self-rated health was significantly associated with perceived environmental hazards. Through the ordered multivariate logistic regression model, we reveal the effects of sociodemographic characteristics and geographical context on health and environmental inequalities in Zhengzhou City. The main findings of this study are as follows: (1) Overall, most residents assessed their health status as good in Zhengzhou City. Among the four perceived environmental hazards, they considered that air pollution and landfill pollution were more serious than noise and water pollution. (2) The self-rated health and four perceived environmental hazards showed significant spatial inequality features. People resident in subdistricts in the western and southern areas had lower levels of self-rated health, while a higher proportion of good-health clusters was found in the eastern and northern areas. Regarding perceived environmental hazards, subdistricts with higher exposure to air pollution and noise pollution were revealed in the northwestern and southern areas; subdistricts with more landfills and water pollution were mainly spread in the northern and southern marginal areas of Zhengzhou City. In addition, our findings also reveal that the clusters of good self-rated health across the entire city had favorable environmental quality where high levels of environmental hazards were located. (3) Residents with different socioeconomic features had different perceptions of health status and environmental hazards. Females had a higher proportion of self-rated good health than males, and the unmarried status of residents corresponded with better health conditions than those of married residents. There was positive correlation among income level, educational attainment, and health conditions, whereby people with high levels of education and income had a better perception of their health condition than those with low levels of education and income. (4) Environmental hazards were among the main factors affecting the self-rated health status of residents, and people who perceived lower exposure to environmental hazards (noise pollution, air pollution, landfill pollution, and water pollution) were more likely to suggest good health conditions. Among these four environmental hazards, water pollution and noise pollution had the greatest impacts on resident’s self-rated health status. In addition, our findings also reveal that socially vulnerable groups reported higher exposure to environmental hazards than others. The perceived high exposure to four environmental hazards was biased toward low-income groups. (5) From the relationship between self-rated health and geographical context, residents with better exposure to green coverage generally reported higher levels of self-rated health condition, while those who perceived worse urban waterlogging reported poorer self-rated health conditions. In addition, we reveal that the geographical location of a subdistrict also had a significant impact on the difference in residents’ self-rated health status.

Our research study focuses on combining multiple approaches to investigate inequalities in health and environmental hazards within a geographical context. These findings provide useful insights on the existing social vulnerability of health and environmental inequalities and help to improve existing environmental policies. Policy makers do need to enact a definite plan to integrate public health, environmental protection, and social equality into the governmental management system and find a balance between the efficiency and equity of environmental and public health at a fine scale.

## Figures and Tables

**Table 1:** The statistics of key sociodemographic variables.

Variables	Variable Description and Proportion (%)
Age	18–29 (26.52%); 30–39 (31.39%); 40–49 (23.82%); 50–59 (7.63%); ≥60 (10.65%)
Gender	Male (56.65%); Female (49.35%)
Marital status	Married (77.12%); Unmarried (21.94%); Others (0.94%)
Education	Primary (4.76%); Secondary (14.21%); Tertiary (78.58%); Postgraduate (2.45%)
Monthly income (RMB)	<1400 (0.05%); 1401–2000 (5.05%); 2001–3000 (24.6%); 3001–6000 (36.62%); ≥6000 (33.68%)
Residence status (hukou)	Local resident (80.17%); Migrant (19.83%)
Housing type	Commodity housing (64.29%); Rented housing (32.69%); Danwei housing (3.02%)

**Table 2:** Multivariate linear regression results of residents’ subjective health perception.

Variable	Model 1		Model 2		Model 3	
	Estimate	t-Value	Estimate	t-Value	Estimate	t-Value
**Gender (Contrast: Male)**						
Female	−0.12077	−1.9774 **	−0.13947	−2.27109 **	−0.15128	−2.40575 ***
**Age (Contrast: 18–29)**						
30−39	−0.0441	−0.4179	−0.08685	−0.81673	−0.12214	−1.11503
40−49	−0.08983	−0.7652	−0.06144	−0.51945	−0.06615	−0.54683
50−59	0.02916	0.1954	0.00164	0.01087	−0.04484	−0.29049
≥60	−0.0191	−0.1319	−0.03996	−0.27367	−0.06954	−0.46445
**Education (Contrast: Primary)**						
Secondary	−0.29171	−1.7941 **	−0.39079	−2.38616 ***	−0.42781	−2.56082 ***
Tertiary	−0.53459	−3.4963 ***	−0.58486	−3.7998 ***	−0.60226	−3.81043 ***
Postgraduate	−0.91998	−3.7491 ***	−0.93109	−3.76829 ***	−0.87297	−3.45236 ***
**Marital status (Contrast: Married)**						
Unmarried	−0.12725	−1.1775	−0.18276	−1.67121 **	−0.20967	−1.86258 **
Others	0.80207	2.4182 ***	0.7871	2.3532 **	0.654995	1.93277 **
**Residence status (Contrast: Local resident)**						
Migrant	−0.11696	−1.1261	−0.09181	−0.87303	−0.14167	−1.31027 *
**Monthly income (Contrast:** **<1400 RMB)**						
1401−2000	−2.58694	−2.2399 **	−2.75676	−2.34506	−2.79142	−2.34479 **
2001−3000	−2.98865	−2.5973 ***	−3.15074	−2.6879 ***	−3.20708	−2.69903 ***
3001−6000	−2.90663	−2.5283 ***	−3.05599	−2.60787	−3.1501	−2.65216 ***
>6000	−2.78342	−2.4211 ***	−2.90936	−2.4825 ***	−3.01443	−2.53742 ***
**Walking distance to the nearest hospital (Contrast:** **<1 km)**						
1−3 km	−0.0443	−0.6757	−0.05878	−0.89005	−0.09188	−1.34569 *
≥3 km	−0.31986	−2.2543 **	−0.36723	−2.55879 ***	−0.37193	−2.4985 ***
**Greening coverage (Contrast: Verygood)**						
Good	0.64887	6.7276 ***	0.33796	3.25761 ***	0.357945	3.38118 ***
Fair	1.22326	11.5627 ***	0.78936	6.83316 ***	0.790332	6.70318 ***
Bad	1.41848	9.9129 ***	0.93469	6.08199 ***	0.903472	5.76069 ***
Very bad	0.54758	1.3017 *	0.29634	0.68471	0.378306	0.85676
**Housing type (Contrast: Commodity housing)**						
Rented housing	−0.09131	−0.9317	−0.11341	−1.14072	−0.11477	−1.13111
*Danwei* housing	−0.0773	−0.4006	−0.1645	−0.84956	−0.22403	−1.13063
**Housing area (Contrast: Housing area** **<** **100 m^2^)**						
Housing area ≥ 100 m^2^	−0.51824	−6.7257 ***	−0.51184	−6.60108 ***	−0.50503	−6.27832 ***
**Urban waterlogging (Contrast: very good)**						
good	−0.09806	−1.0205	−0.16669	−1.62373 *	−0.17909	−1.69718 **
Fair	0.15536	1.5962 *	0.03447	0.33777	0.006172	0.05892
bad	0.54859	4.8724 ***	0.25674	2.0679 **	0.237911	1.8784 **
Very bad	0.61814	3.2046 ***	0.35968	1.71195 **	0.324823	1.51901 *
**Water pollution (Contrast: Very low)**						
Low			0.34993	3.16219 ***	0.36494	3.23853 ***
Fair			0.56717	5.00739 ***	0.571593	4.9426 ***
High			0.29573	2.02868 **	0.321115	2.16455 **
Very high			0.45921	2.07983 **	0.564549	2.48986 ***
**Landfill pollution (Contrast: Very low)**						
Low			0.18769	1.65067 **	0.183207	1.57489 *
Fair			0.31574	2.83984 ***	0.306699	2.69744 ***
High			0.49848	3.77843 ***	0.444475	3.29695 ***
Very high			0.52889	2.55582 ***	0.515924	2.44589 ***
**Air pollution (Contrast: Very low)**						
Low			0.37653	3.27875 ***	0.399906	3.40899 ***
Fair			0.34572	3.02955 ***	0.394031	3.37957 ***
High			0.24951	1.94122 **	0.286432	2.18339 **
Very high			0.03808	0.19984	0.111245	0.57333
**Noise pollution (Contrast: Very low)**						
Low			0.24434	2.217 **	0.252614	2.24819 **
Fair			0.4766	4.21765 ***	0.490912	4.27303 ***
High			0.5428	4.06375 ***	0.554227	4.07914 ***
Very high			0.36328	1.70501 **	0.422682	1.94788 **
**Subdistrict (*Jiedao*) (Contrast: Sanguanmiao Subdistrict)**						
Chengdonglu Subdistrict					1.00996	2.75323 ***
Huayuankou town					−0.9158	−1.76298 **
Dongdajie Subdistrict					0.828687	2.34175 **
Lvdongcun Subdistrict					1.033971	2.47699 ***
Longyuanlu Subdistrict					1.183477	1.72045 *
Erligang Subdistrict					0.828864	2.15024 **

Note: significance level: *** *p* < 0.01, ** *p* < 0.05, * *p* < 0.1.

## Data Availability

Not applicable.
